# Virulence factor RNA transcript expression in the *Leishmania Viannia* subgenus: influence of species, isolate source, and *Leishmania* RNA virus-1

**DOI:** 10.1186/s41182-019-0153-x

**Published:** 2019-04-11

**Authors:** Ruwandi Kariyawasam, Avinash N. Mukkala, Rachel Lau, Braulio M. Valencia, Alejandro Llanos-Cuentas, Andrea K. Boggild

**Affiliations:** 1 Institute of Medical Sciences, University of Toronto, Toronto, ON Canada; 2 Public Health Ontario Laboratory, Toronto, ON Canada; 3 Instituto de Medicina Tropical “Alejandro von Humboldt”, Lima, Peru; 4 Facultad de Salud Pública y Administración, Universidad Peruana Cayetano Heredia, Lima, Peru; 5 Department of Medicine, University of Toronto, Toronto, ON Canada; 6 Tropical Disease Unit, Toronto General Hospital, 200 Elizabeth Street, 13EN-218, Toronto, ON M5G 2C4 Canada; 7 Viral Immunology Systems Program, Kirby Institute, University of New South Wales, Sydney, Australia

**Keywords:** American tegumentary leishmaniasis, *Leishmania Viannia braziliensis*, *Leishmania* RNA Virus-1 (LRV1), Virulence factor

## Abstract

**Background:**

*Leishmania* RNA virus-1 (LRV1) is a double-stranded RNA virus identified in 20–25% of *Viannia*—species endemic to Latin America, and is believed to accelerate cutaneous to mucosal leishmaniasis over time. Our objective was to quantify known virulence factor (VF) RNA transcript expression according to LRV1 status, causative species, and isolate source.

**Methods:**

Eight cultured isolates of *Leishmania* were used, four of which were LRV1-positive (*Leishmania Viannia braziliensis* [*n* = 1], *L*. (*V*.) *guyanensis* [*n* = 1], *L*. (*V*.) *panamensis* [*n* = 2]), and four were LRV1-negative (*L*. (*V*.) *panamensis* [*n* = 3], *L*. (*V*.) *braziliensis* [*n* = 1]). Promastigotes were inoculated into macrophage cultures, and harvested at 24 and 48 h. RNA transcript expression of *hsp23*, *hsp70*, *hsp90*, *hsp100*, *mpi*, *cpb*, and *gp63* were quantified by qPCR.

**Results:**

RNA transcript expression of *hsp100* (*p* = 0.012), *cpb* (*p* = 0.016), and *mpi* (*p* = 0.022) showed significant increases from baseline pure culture expression to 24- and 48-h post-macrophage infection, whereas *hsp70* (*p* = 0.004) was significantly decreased. A trend toward increased transcript expression of *hsp100* at baseline in isolates of *L*. (*V*.) *panamensis* was noted. Pooled VF RNA transcript expression by *L*. (*V*.) *panamensis* isolates was lower than that of *L*. (*V*.) *braziliensis* and *L*. (*V*.) *guyananesis* at 24 h (*p* = 0.03). VF RNA transcript expression did not differ by LRV1 status, or source of cultured isolate at baseline, 24, or 48 h; however, a trend toward increased VF RNA transcript expression of 2.71- and 1.93-fold change of *mpi* (*p* = 0.11) and *hsp90* (p = 0.11), respectively, in LRV1 negative isolates was noted. Similarly, a trend toward lower levels of overall VF RNA transcript expression in clinical isolates (1.15-fold change) compared to ATCC® strains at 24 h was noted (*p* = 0.07).

**Conclusions:**

Our findings suggest that known VF RNA transcript expression may be affected by the process of macrophage infection. We were unable to demonstrate definitively that LRV-1 presence affected VF RNA transcript expression in the species and isolates studied. *L*. (*V*.) *guyanensis* and *L*. (*V*.) *braziliensis* demonstrated higher pooled VF RNA transcript expression than *L*. (*V*.) *panamensis*; however, further analyses of protein expression to corroborate this finding are warranted.

**Electronic supplementary material:**

The online version of this article (10.1186/s41182-019-0153-x) contains supplementary material, which is available to authorized users.

## Background

American tegumentary leishmaniasis (ATL) is comprised of cutaneous leishmaniasis (CL), mucocutaneous leishmaniasis (MCL), and mucosal leishmaniasis (ML), which are endemic to Central and South America [1]. Transmitted by sandflies, the array of clinical manifestations depends on the *Leishmania* spp. involved as well as the immunological status of the host [2, 3]. The outcomes of infection greatly depend on host and parasitological factors whereby the protozoan parasite gains access to the host cell, and survives by either suppressing or evading the host immune response [4, 5]. While most CL presents as a painless ulcer, in particular, the *Viannia* subgenus has been implicated in severe disease including inflammatory CL, characterized by erythema, purulent exudate, pain, and/or lymphatic involvement (“complex” as per the Infectious Diseases Society of America (IDSA) cutaneous leishmaniasis guidelines), in addition to MCL and ML [6]. Parasitological factors known to modulate the host immune response include *Leishmania* RNA virus-1 and endogenous virulence factors.

A double-stranded RNA virus, *Leishmania* RNA virus-1 (LRV1), has been identified in certain strains of the *Viannia* species predominantly found in the Amazon basin of South America [7, 8]. Geographical expansion as a result of environmental changes and urbanization is postulated to have caused the parasite-harboring LRV1 to spread to Central America [9, 10]. LRV1 has been associated with an over-active immune response with increased expression of proinflammatory cytokines and chemokines including TNF-α, IL-6, CXCL10, CCL4, CCL5, and is believed to accelerate 10–15% of localized CL to either ML or MCL [11–13]. To add, LRV1 has been documented in 20–58% of clinical isolates of *L*. (*V*.) *guyanensis* and *L*. (*V*.) *braziliensis* associated with first-line treatment failure and relapse [14, 15].

Virulence factors (VFs) are molecules that enable pathogen adaptation to adverse environmental conditions through increased expression, or via manipulation of the host immune response [4, 5, 16]. VFs endogenous to *Leishmania* spp. including molecular chaperones such as heat-shock proteins (HSPs), cysteine proteinases (CPB), leishmanolysins, phosphatases, and proteinases have been known to aid in the promastigote-amastigote transformation process and have certain immunomodulatory effects [4, 17, 18].

The current paradigm is that most genes of *Leishmania* are constitutively expressed, with fewer than 5% of mRNA transcripts varying significantly between life cycle stages [19–22]. Thus, regulation of gene control is thought to occur post-transcriptionally, and even post-translationally, and as such, the transcriptome has been thought of as a poor correlate of protein expression [23–25]. Host immune pressure has also been thought not to affect parasite gene expression at the RNA level [25]. Recent transcriptomic approaches using next-generation sequencing suggest a possible correlation between RNA abundance and ultimate protein expression, even in genes known to be constitutively expressed [26]. For example, HSP70 accounts for > 2% of total protein in *Leishmania* promastigotes, and similarly, *hsp70* transcripts correspond to two of the top three most abundant transcripts in promastigotes of *L*. *major* [26]. Moreover, 20 of the 50 most abundant transcripts encode ribosomal proteins [26]. In the meta-transcriptome profiling analysis of human *L*. (*V*.) *braziliensis* infection, Christensen and colleagues suggest that RNA transcripts identified in CL clinical lesions might be those contributing to promotion of parasite persistence, rather than just those of the most highly expressed *Leishmania* genes [27]. Such findings may suggest that in human *L*. (*V*.) *braziliensis* infection, at least, those proteins contributing to parasite subversion of the host clearance machinery are, indeed, correlated to corresponding RNA transcript levels. Furthermore, that many of the most abundant RNA transcripts encode putative proteins of as-yet undetermined function [25–27] underscores that our understanding of how the parasite transcriptome might correlate to the functional proteome and ultimate virulence and pathogenesis of *Leishmania* remains an area with knowledge gaps to be filled. It has been documented that among CL lesions due to *L*. (*V*.) *braziliensis*, there is high uniformity of RNA transcript expression regardless of lesion size and duration [27]. Thus, we aimed to ascertain the relative abundance of known VF RNA transcripts, including *hsp23*, *hsp70*, *hsp90*, *hsp100*, *cpb*, zinc metalloproteinase GP63 (*gp63*), and mannose phosphate isomerase (*mpi*), previously evaluated in Old World strains and for which sequences were readily available through the National Center for Biotechnology Information (NCBI) database, in pure cultures and a macrophage model of infection with several species of the *Leishmania Viannia* subgenus, a group around which few such data exist. In addition, we aimed to understand the influence, if any, of isolate source, corresponding species, and LRV1 status on VF RNA transcript expression and further comment on the virus, parasite, and host dynamics in regards to infection.

## Methods

### Ethics approval

Approval for this study was obtained from the Ethics Review Board of Public Health Ontario.

### Clinical data

De-identified clinical data of source patients collected from test requisitions were stratified into “severe” and “non-severe” phenotypes as per the IDSA guidelines [6], where a severe phenotype was defined as mucosal involvement; ulcers with associated erythema, purulent exudate, pain, and/or lymphatic involvement (inflammatory ulcers); or multifocal/disseminated disease (ulcers in ≥ 2 anatomic sites and ≥ 4 in number) (Table 1) [6]. A non-severe phenotype was defined as localized CL (LCL) of < 4 ulcers in number [6] (Table 1).

**Table 1:** Classification of *Leishmania* spp. strains used in experiments

*Leishmania* Strain	Source of isolate	Country of acquisition	LRV1 status	Relative LRV1 copy number	Age	Sex	Clinical phenotype
*L*. (*V*.) *braziliensis* (LVb−)	ATCC (MHOM/BR/75/M2903)	Brazil	LRV1−	NA	Unk	Unk	Unk
*L*. (*V*.) *braziliensis* (LVb+)	Clinical	Peru	LRV1+	0.1	22	Male	Severe
*L*. (*V*.) *guyanensis* (LVg+)	ATCC (MHOM/BR/75/M4147)	Brazil	LRV1+	Reference [1]	Unk	Unk	Unk
*L*. (*V*.) *panamensis* (LVp0−)	ATCC (MHOM/PA/71/LS94)	Unknown	LRV1−	NA	Unk	Unk	Unk
*L*. (*V*.) *panamensis* (LVp1−)	Clinical	Costa Rica	LRV1−	NA	80	Male	Severe
*L*. (*V*.) *panamensis* (LVp2−)	Clinical	Costa Rica	LRV1−	NA	17	Male	Non-severe
*L*. (*V*.) *panamensis* (LVp1+)	Clinical	Ecuador	LRV1+	2.17 × 10^−4^	9	Male	Severe
*L*. (*V*.) *panamensis* (LVp2+)	Clinical	Costa Rica	LRV1+	1.02 × 10^−4^	71	Male	Severe

### Cultured *Leishmania* spp.

#### *Leishmania* strains

Relevant characteristics of each of the *Leishmania* strains used are summarized in Table 1. Cultured isolates of *Leishmania* were obtained from the American Type Culture Collection® (ATCC®), and our *Leishmania* biobank of surplus cultured isolates at Public Health Ontario Laboratories (PHOL) as previously described [28]. The following species of *Leishmania* were used: ATCC® strains of *L*. (*V*.) *braziliensis* ATCC®50135™ (MHOM/BR/75/M2903) LRV1 negative (LVb−); *L*. (*V*.) *guyanensis* ATCC®50126™ (MHOM/BR/75/M4147) LRV1+ (LVg+); *L*. (*V*.) *panamensis* ATCC®50158™ (MHOM/PA/71/LS94) LRV1 negative (LVp0−); and five clinical strains including one LRV1 positive *L*. (*V*.) *braziliensis* (LVb+), four *L*. (*V*.) *panamensis* including two LRV1 negative *L*. (*V*.) *panamanensis* (LVp1− and LVp2−), and two LRV1 positive *L*. (*V*.) *panamensis* (LVp1+ and LVp2+) (Table 1). Promastigotes were routinely subcultured in Tobie’s medium with Locke’s overlay at ambient temperature every week. The following passage numbers (P#) of ATCC® and clinical isolates were used in this study: P2 (LVg+ and LVp2−), P3 (LVp2+), P5 (LVp1+), P6 (LVb−), P7 (LVb+), and P8 (LVp0− and LVp1−) (Table 1). Prior to macrophage infection, 1.6 mL of *Leishmania* promastigotes was obtained and stored in − 80 °C to be used for species molecular identification, LRV1 detection, and quantification.

#### Macrophage infection

Macrophages were infected with promastigotes at a multiplicity of infection (MOI) of 10:1 (parasite: macrophage) in triplicate (see Additional file 1 for macrophage differentiation). Prior to infection, a cell count of the *Leishmania* promastigotes was performed. Prior to addition to the 24-well plates, promastigotes were centrifuged at 1000 rpm for 5 min and the cell pellet was resuspended with fresh Roswell Park Memorial Institute (RPMI) 1640 medium supplemented with 10% fetal bovine serum (FBS) [28]. Subsequently, the plates were placed in an incubator set at 37 °C and 5% CO_2_ [28]. Supernatants of infected macrophages adhering to the coverslips containing amastigotes were released using 0.05% Trypsin-ethylenediaminetetraacetic acid (EDTA) (Life Technologies, Carlsbad, CA, USA), collected at 24 and 48 h and were stored in − 80 °C until downstream RNA extraction, cDNA synthesis and VF RNA transcript expression analysis.

### VF RNA transcript expression and LRV1 quantification

#### RNA extraction for VF transcript expression and determination of LRV1 status

RNA was extracted from baseline pure culture and infected macrophages released using 0.05% Trypsin-EDTA, using QIAmp RNA Mini Kit (Qiagen, Germantown, MA, USA). An in-column DNase treatment was included in all extractions as per manufacturer’s protocol.

#### cDNA synthesis

cDNA was synthesized with 50–300 ng of RNA using Superscript II Reverse Transcriptase and random hexamers (Life Technologies, Carlsbad, CA, USA), followed by purification with QIAquick PCR Purification Kit (Qiagen, Germantown, MA, USA), and eluted with 50 μL of nuclease-free water. In cases where RNA level was too low, a pre-amplification reaction to increase sensitivity of detection was performed with Perfecta Pre-Amp Supermix (Quanta Biosciences, Gaithersburg, MD, USA) using 100 ng of cDNA according to manufacturer’s protocol with 14 cycles. The reaction was diluted 1:20 and 5 μL of the pre-Amp cDNA was used in subsequent qPCR as above.

#### Detection of LRV1 by qPCR on baseline pure culture

Two real-time PCR (qPCR) assays for detection of LRV1 were performed with LRV1 set A and set B primers respectively as previously described [29]. *Leishmania* kinetoplastid membrane protein 11 (*kmp11*) was used as a quantification and extraction control [29]. Sybr Green real-time PCR was setup with 1x Sybr Select Master Mix (Life Technologies, City, State), 250 nM final concentration of forward and reverse primers, 5 μL of cDNA of pure culture in a total volume of 20 μL [29]. Amplification was performed in an ABI 7900HT real-time instrument with the following conditions: uracil-DNA glycosylase (UDG) activation at 50 °C for 2 min, polymerase activation at 95 °C for 2 min, followed by 45 cycles of 95 °C for 15 s, and 60 °C for 1 min. A dissociation step of 95 °C for 15 s, 60 °C for 15 s, and again another 95 °C for 15 s was added at the end to generate a melting curve, which was used to check for the specificity of amplification. ATCC®50126™ *L*. (*V*.) *guyanensis* strain MHOM/BR/75/M4147, known to be LRV1 positive, was used as a positive control and RNA from a healthy human individual as negative control. LRV1 was confirmed by melt-curve analysis and was quantified relative to kinetoplastid membrane protein 11 (kmp11) and ATCC®50126™ *L*. (*V*.) *guyanensis* using the 2^-∆∆Ct^ method [30].

#### Detection and quantification of VF RNA transcript expression by qPCR

RNA transcript expression of the following virulence factors: *hsp23*, *hsp70*, *hsp90*, *hsp100*, *mpi*, *cpb*, *gp63*, and 18S, was performed on the ABI 7900HT real-time instrument using primers designed using Primer Express 3.0.1 (ThermoFisher Scientific, Carlsbad, CA, USA) (Table 2). A real-time PCR was set up in triplicate using 12.5 μL 2x Taqman Universal Master Mix (ThermoFisher Scientific, Carlsbad, CA, USA), 250 nM final concentration of forward and reverse primers, 10 nM probe, and 5 μL of cDNA from baseline pure culture or post-macrophage infection at 24 or 48 h, in a total volume of 20 μL for each respective target (Table 2) [29]. Amplification was performed with the following conditions: UDG activation at 50 °C for 2 min, polymerase activation at 95 °C for 10 min, followed by 45 cycles of 95 °C for 15 s and 60 °C for 1 min. Virulence factor RNA transcript expression was quantified relative to 18S of each culture using the 2^-∆Ct^ method [30].

**Table 2:** Primer and probe sequences used to detect virulence factor RNA transcripts by real-time PCR

Target	Sequence
18S	
Forward	5′-AAGTGCTTTCCCATCGCAACT-3′
Reverse	5′-GACGCACTAAACCCCTCCAA-3′
Probe	FAM-CGGTTCGGTGTGTGGCGCC-NFQ
GP63	
Forward	5′-GGCTTCTACCAGGCGGACTT-3′
Reverse	5′-TGATGY^b^Y^b^BTBCR^a^CCATGCACTT-3′
Probe	FAM-AGGCCGAGGTGATG-MBG
CPB	
Forward	5′-GCTCGTCGGGTACAACAAGAC-3′
Reverse	5′-AGTCCTCACCCCACGAGTTCT-3′
Probe	FAM-TTCCGTACTGGGTGATC-BHQ1
MPI	
Forward	5′-GCTGCGAGGCCGGATAA-3′
Reverse	5′-GGAGTCAAGGCGCAR^a^ATGAG-3′
Probe	FAM-TACAAGGACCCGAACCACAR^a^GCCTGA-BHQ1
HSP23	
Forward	5′-GAR^a^CGS^c^TGCTTCGAGCTT-3′
Reverse	5′-GAAGS^c^TGGCCTTGATTTTGC-3′
Probe	FAM-CTGTTCGAGCTTC-BNFQ
HSP70	
Forward	5′-GTGGAW^d^ATCATCGCGAACGA-3′
Reverse	5′-GAGTCCGTGAACGCAACGTA-3′
Probe	FAM-AGGGY^b^AACCGCACGACACCGT-BHQ1
HSP90	
Forward	5′-CAAGAAGCGCAACAACATCAA-3′
Reverse	5′-TCGCAGTTGTCCATGATGAAC-3′
Probe	FAM-TGTACGTGCGCCGCG-BHQ1
HSP100	
Forward	5′-CCGACTTCCAR^a^GACGACAAC-3′^a^
Reverse	5′-GCCTGCTTGCAGAGATCR^a^A-3′
Probe	FAM-ACGAGTCACTGAACAAG-BHQ1

^a^R = A,G

^b^Y = C,T

^c^S = C,G

^d^W = A,T

### Statistical analysis

**Fig. 1:**
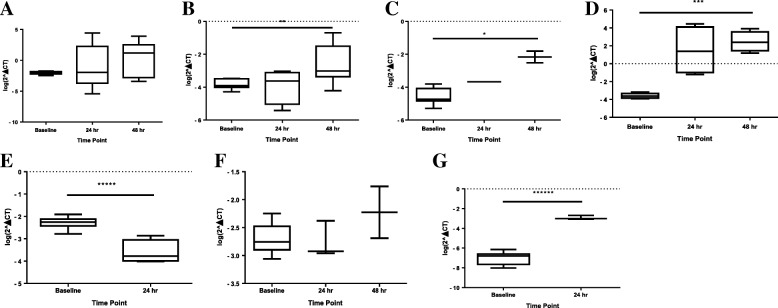
Log transformed virulence factor RNA transcript expression in baseline cultures and supernatants post-macrophage infectivity at 24 and 48 h compared by Kruskal-Wallis or Mann-Whitney for the following targets: pooled expression (**a**), *cpb* (**b**), *gp63* (**c**), *mpi* (**d**), *hsp70* (**e**), *hsp90* (**f**), and *hsp100* (**g**)

Descriptive statistics were performed on clinical data related to the surplus clinical strains housed in the *Leishmania* biobank, including age, sex, travel region, and clinical phenotype. Relative and pooled virulence factor RNA transcript expression was calculated for each strain, compared by species, LRV1 status, and source of cultured isolate (ATCC® versus clinical), using Mann-Whitney *U* Test and Kruskal-Wallis test at baseline pure culture, 24, and 48 h time points post macrophage infection. For the comparison by species, we compared *L*. (*V*.) *panamensis* versus *L*. *V*. *braziliensis* and *L*. *V*. *guyanensis*, based on the premise that *L*. (*V*.) *braziliensis* and *L*. (*V*.) *guyanensis* are generally thought of as more virulent strains manifesting more severe clinical sequelae [6, 11–13]. Pooled virulence factor RNA transcript expression was calculated to determine if expression was enhanced or downregulated due to singular or multi-transcriptional genes. A log transformation was performed to graphically represent the data (Fig. 1, Additional file 2: Figures S1–S6). All statistical analyses were conducted using GraphPad Prism 6 version 6.07 software (GraphPad Software Inc., La Jolla, CA).

## Results

### LRV1 status of isolates

Three of five (60%) clinical cultures were LRV1 positive (LVb+, LVp1+, and LVp2+) and two were LRV1 negative (LVp1− and LVp2−) (Table 1). One of three ATCC® strains was LRV1 positive (LVg+) and two were LRV1 negative (LVb− and LVp0−) (Table 1). Of four cultured clinical isolates from patients with a severe clinical phenotype, all but one were LRV1 positive (Table 1). Relative LRV1 copy number of positive isolates ranged from 2.17 × 10^−4^ to 0.10 copies/mL (Table 1).

### Virulence factor RNA transcript expression

Baseline virulence factor transcript expression of all targets was detected in the eight cultures. The following targets could not be evaluated in post-macrophage infectivity supernatants due to transcript levels below detection: *gp63* (24 h), *hsp23* (24 and 48 h), *hsp70* (48 h), and *hsp100* (48 h). Overall, virulence factor (VF) transcript expression did not differ between baseline pure culture, 24-, and 48-h post-macrophage infectivity for *hsp90* (*p* = 0.40) and pooled VF (*p* = 0.78) analyses (Fig. 1). A significant increase in transcript expression from baseline pure culture to 24 and 48 h was observed for the following VF transcripts: *cpb* (*p* = 0.016), *mpi* (*p* = 0.022), *gp63* (*p* = 0.044), and *hsp100* (*p* = 0.012) (Fig. 1). A significant decrease in transcript expression of *hsp70* by 31.4-fold was observed between baseline pure culture and 24-h post macrophage infectivity (*p* = 0.004) (Fig. 1).

### VF RNA transcript expression by LRV1 status

VF transcript expression did not differ by LRV1 status for all baseline pure *Leishmania* cultures (pre-macrophage infection) for *gp63* (*p* = 0.20), *cpb* (*p* = 0.49), *hsp23* (*p* = 0.34), *hsp70* (*p* = 0.34), *hsp100* (*p* = 0.34), and pooled (*p* = 0.34) analyses (Additional file 2: Figure S1). A trend toward a 2.71- and 1.93-fold increased transcript expression of *mpi* (*p* = 0.11) and *hsp90* (p = 0.11), respectively, in LRV1 negative isolates was noted (Additional file 2: Figure S1). VF RNA transcript expression by LRV1 status across time points is presented in Additional file 1 (Additional file 2: Figure S2).

### VF RNA transcript expression by species

VF transcript expression did not differ by species (*L*. (*V*.) *panamensis* versus *L*. (*V*.) *braziliensis* and *L*. (*V*.) *guyanensis*) for all baseline pure *Leishmania* cultures (pre-macrophage infection) for the following: *gp63* (*p* = 0.50), *cpb* (*p* = 0.25), *mpi* (*p* = 0.86), *hsp23* (*p* = 0.68), *hsp70* (*p* = 0.79), *hsp90* (*p* = 0.50), and pooled (*p* > 0.99) analyses (Additional file 2: Figure S3). Increased transcript expression of *hsp100* in isolates of *L*. (*V*.) *panamensis* was noted; however, this was not statistically significant (*p* = 0.14) (Additional file 2: Figure S3). Pooled VF transcript expression by *L*. (*V*.) *panamensis* isolates was lower than that of *L*.(*V*.) *braziliensis* and *L*. (*V*.) *guyananesis* at 24 h (*p* = 0.03) (Additional file 1, Additional file 2: Figure S4).

### VF RNA transcript expression by source of cultured isolate

VF transcript expression did not differ by source of cultured isolate (ATCC® versus clinical) for all baseline pure *Leishmania* cultures (pre-macrophage infection) for the following: *gp63* (*p* = 0.74), *cpb* (p = 0.79), *mpi* (p = 0.79), *hsp23* (p = 0.74), *hsp70* (*p* = 0.68), *hsp90* (*p* = 0.79), *hsp100* (*p* = 0.57), and pooled (*p* = 0.86) analyses (Additional file 2: Figure S5). VF RNA transcript expression by source of cultured isolate across time points is presented in Additional file 1 (Additional file 2: Figure S6). At 24 h, there was a trend toward lower levels of overall VF transcript expression in clinical isolates (1.15-fold change) compared to ATCC® strains (*p* = 0.07) (Additional file 2: Figure S6).

## Discussion

Numerous parasitological factors enhance the ability of the *Leishmania* parasite to produce a successful infection, including infecting species, parasite load, LRV1 status, and most importantly, the expression of virulence factors [2–4, 6]. Many studies have separately evaluated the role of LRV1 [7–9, 11–13] and VFs [16, 31–35] in the pathogenesis of ATL; however, data on the combined role of both factors in ATL pathogenesis are scarce. We evaluated the contribution of LRV1 to key VF RNA transcript expression in the *Viannia* subgenus given its role as a mammalian host immunomodulator and potential influence on parasite itself, and did not demonstrate any change in relative abundance of VF RNA transcripts based on LRV1 status. However, there was a trend toward an almost two- and threefold increased transcript expression of *hsp90* and *mpi*, respectively, in LRV1 negative isolates. Overall, we noticed significant differential VF transcript expression resulting from pure cultures to the macrophage model, and also noted an overall reduction in VF transcript expression in isolates of *L*. (*V*.) *panamensis* compared to other *Vianna* strains, with a trend in decreasing expression of *hsp100* in *L*. (*V*.) *panamensis*. Differential RNA transcript expression by source of the cultured isolate was also not observed. This is also one of few studies to document LRV1-positive *L*. *V*. *panamensis* isolates from Central America [36].

Molecular chaperones are key proteins involved in the maintenance of cellular homeostasis through folding of polypeptides [31]. Heat shock proteins are a subset of molecular chaperones known to increase in synthesis when presented with heat stress [31, 32]. HSP23 is preferentially expressed up to threefold in the mammalian stage for *Leishmania* infectivity of macrophages, and is essential for stress tolerance and implicated in protection against trivalent antimonials [32]. HSP70 is the most conserved protein present in all eukaryotes and is involved in cell survival through avoidance of protein denaturation, and is often coupled with HSP90 [32, 33]. HSP90, the most abundant protein in eukaryotic cytoplasm, mitochondria, and endoplasmic reticulum, is involved in the maintenance of numerous kinases and transcription factors [32]. HSP90 is also implicated in the maturation of viral proteins [32]. We found a trend toward higher expression of *hsp90* in LRV1 negative isolates compared to LRV1 positive isolates, which contravenes *hsp90*’s role in viral protein maturation. Subsequent protein work would be necessary to corroborate this finding. Lastly, HSP100 works in association with HSP70 to recognize misfolded proteins and is often an antagonist to the transformation of the amastigote back to the promastigote stage in *L*. *donovani* [31–34]. We noted a trend toward lower expression of *hsp100* in isolates of *L*. (*V*.) *panamensis* compared to *L*. (*V*.) *braziliensis* and *L*. (*V*.) *guyanensis*, which might simply reflect that one strain of *L*. (*V*.) *panamensis* originated from a patient with non-severe CL. HSPs have also been involved in altering the immune response to *Leishmania* infection, whereby adjuvant effects of HSPs were observed in mice infected with *L*. *major* and were shown to induce IL-1, IL-6, IL-12, and TNF-α expression contributing to a Th1 cytokine pattern of cellular immunity [35]. Overall, our data supported an increased RNA transcript expression of *hsp100* upon transformation of promastigotes to amastigotes during macrophage infection. Interestingly, we observed a significant decrease in *hsp70* transcript expression upon macrophage infection at 24 h, with no commensurate difference in *hsp90* transcript expression, countering the observed paradigm of HSP70 and 90 coupling. It has been reported that increased synthesis of *hsp70* or *hsp90* transcripts does not correlate to increases in levels of proteins significantly, thus the reduction in *hsp70* transcripts observed in our study may not affect protein levels observed post-macrophage infection [41, 42]. Future disentanglement of how the *hsp70-hsp90* RNA transcript relationship might translate to protein expression and coupling in the in vivo situation is warranted.

Cysteine peptidases (CPs) are virulence factors present in all pathogenic kinetoplastida, and are considered potential therapeutic and vaccine candidates given their ability to modulate host-parasite interactions [37, 38]. Three distinct genes exist: CPA, CPB, and CPC, all belonging to the same group designated Clan CA, Family C1 [37]. CPB is a key regulator of parasite stage differentiation, and is associated with a Th2 cytokine response by increasing IL-4 production, degrading NF-κβ and IL-12, thereby dampening the Th1 cytokine response [6, 37, 39]. Our data revealed an increase in expression of *cpb* transcripts from baseline to post-macrophage infection at 24 and 48 h, and such a response, assuming correlation of RNA and protein levels, may correlate with disease severity through dampening of a Th1-directed cytokine profile and immunologic response to infection, which has been demonstrated in previously published in vivo models [6, 37, 39]. Further evaluation of such a hypothesis using human skin models and human PBMCs is warranted.

Metalloproteases such as the zinc-dependent metalloprotease, glycoprotein 63, is a major surface antigen expressed on all *Leishmania* spp. promastigotes, and is involved in parasite adherence to macrophages and evasion of complement-mediated lysis [3, 6, 16]. GP63 activates protein tyrosine phosphatases (PTPs) to reduce nitric oxide (NO) production, thus facilitating parasite persistence in the macrophage vacuole [40]. Our data revealed an increase in *gp63* transcript expression from baseline culture to 24- and 48-h post-macrophage infection at levels similar to *cpb* transcript expression. It has been found that CPB is required for GP63 expression, thus allowing the parasite to thrive in the macrophage [38]. Lastly, MPI is an enzyme involved in the reversible conversion of fructose-6-phosphate and mannose-6-phosphate required for biosynthesis of various glycoconjugates [41]. Lack of MPI has been associated with slowed growth in *Leishmania* spp. Our data have demonstrated a significant increase in *mpi* transcript expression from baseline to post-macrophage infection at 24 and 48 h, as well as in LRV1 negative compared to positive isolates, which, assuming correlation of RNA and protein levels, could contribute to maintenance of the parasite’s virulence to colonize host cells.

Our data have provided insight into VF RNA transcript expression in different LRV1-positive and -negative *Viannia* strains causing ATL, including *L*. (*V*.) *guyanensis* and *L*. (*V*.) *panamensis* about which few such data exist. Although we did not observe significant differences in VF transcript expression attributable to source of cultured isolate or LRV1 status, we did observe an overall diminution of VF transcript expression in *L*. (*V*.) *panamensis* compared to the historically more clinically aggressive *L*. (*V*.) *braziliensis* [2, 6–8, 11–13]. The trend toward increased expression of *mpi* and *hsp90* in LRV1-negative isolates is interesting and requires future analyses with more isolates and a focus on protein expression to reconcile the relationship between VF expression, macrophage infection, clinical disease, and LRV1. One possibility would be that in the presence of LRV1, parasites are able to successfully infect macrophages without elaboration of specific VFs, while in its absence, cellular expenditures to produce VF that enhance macrophage infection are required. Further examination of species-specific virulence factors including leishmanolysins may illuminate aspects of infection severity, particularly as seen in *L*. (*V*.) *braziliensis* infection [18]. The host immune response may weigh heavily on the outcome of parasitic infection in addition to select virulence factors where host phosphatases such as serine threonine phosphatases (STPs) have been shown to regulate the outcome of *Leishmania* spp. infection [17]. This latter finding is consistent with the findings of Christensen and colleagues who noted uniform transcript expression across lesions due to *L*. (*V*.) *braziliensis* despite clinical variability, particularly size and lesion duration [27].

## Limitations

Limitations of this work include the small number of cultured isolates from a limited geographic range, as well as differences in the passaging of the strains in order to achieve sufficient promastigote growth phase and concentrations for successful macrophage infection. Additionally, all clinical isolates were derived from male patients, and this may affect the generalizability of the data. Moreover, only three different species of *Leishmania* were found to contain LRV1, thereby limiting our ability to stratify our analyses by both LRV1 status and species. Within clinical cultures known to be LRV1 positive, the possibility that mixed LRV1 positive and negative strains exists, contributing to the lower viral load compared to *L*. (*V*.) *guyanensis* ATCC®50126™ (MHOM/BR/75/M4147). Furthermore, not all VF transcripts were detectable by our assays at all time points, which may have resulted from the limited availability of sequences for which our primers were designed and could have biased our interpretation of the data. It is possible that the expression of certain VF transcripts would be detectable at time points greater than 48-h post-macrophage infectivity, though this premise is countered by the findings of Fernandes and colleagues, who noted maximal differential gene expression within 24 h of macrophage infection, with little host-parasite interactions beyond that time point [43]. We evaluated VF RNA transcript expression and did not quantify protein expression, thus, it is unknown whether or not transcript abundance would correlate to protein abundance. Finally, our macrophage model was derived from U937 cells, which may not represent the human in vivo or PBMC model well.

## Conclusions

We have established a human macrophage model of ATL, infection of which was demonstrated to induce known VF RNA transcript expression. Differential VF transcript expression was attributable to the process of macrophage infection, despite that genes of many known VFs are thought to be constitutively expressed. Infecting species, rather than LRV1 status or source of cultured isolate, was also demonstrated to correlate to differential VF RNA transcript expression. Although trends were identified suggesting that LRV1 may inversely correlate to VF RNA transcript expression, including *mpi* and *hsp90*, further studies focused on protein work post-macrophage infection are needed to corroborate this finding.

## Additional files

Additional file 1: Supplementary Methods and Results. (DOCX 24 kb)

Additional file 2: **Figure S1.** Log transformed virulence factor RNA transcript expression in baseline cultures analyzed by grouping strains according to LRV-1 status compared by Mann-Whitney for the following targets: pooled expression (A), *cpb* (B), *gp63* (C), *mpi* (D), *hsp23* (E), *hsp70* (F), *hsp90* (G) and *hsp100* (H). **Figure S2.** Log transformed virulence factor RNA transcript expression in supernatants post-macrophage infectivity at 24 and 48 h compared by LRV-1 status using t-test for the following targets: *cpb*-24 h (A), *mpi*-24 h (B)*, hsp70*–24 h (C), pooled-48 h (D), *cpb*-48 h (E), and *mpi*-48 h (F). **Figure S3.** Log transformed virulence factor RNA transcript expression in baseline cultures analyzed by grouping strains according to species (*L*. *V*. *panamensis* versus other) compared by Mann-Whitney for the following targets: pooled expression (A), *cpb* (B), *gp63* (C), *mpi* (D), *hsp23* (E), *hsp70* (F), *hsp90* (G) and *hsp100* (H). **Figure S4.** Log transformed virulence factor RNA transcript expression in supernatants post-macrophage infectivity at 24 and 48 h compared by species using t-test for the following targets: pooled VF-24 h (A), *cpb*-24 h (B), pooled VF-48 h (C), and *cpb*-48 h (D). **Figure S5.** Log transformed virulence factor RNA transcript expression in baseline cultures analyzed by grouping strains according to source of cultured isolate (ATCC® versus clinical) compared by Mann-Whitney for the following targets: pooled expression (A), *cpb* (B), *gp63* (C), *mpi* (D), *hsp23* (E), *hsp70* (F), *hsp90* (G) and *hsp100* (H). **Figure S6.** Log transformed virulence factor RNA transcript expression in supernatants post-macrophage infectivity at 24 and 48 h compared by source of cultured isolate using t-test for the following targets: pooled VF-24 h (A), pooled VF-48 h (B), *cpb*-48 h (C), and *mpi*-48 h (D). (DOCX 1229 kb)

## Abbreviations

ATCC®: American Type Culture Collection®
ATL: American Tegumentary Leishmaniasis
CL: Cutaneous Leishmaniasis
CPBs: Cysteine proteinases
CPs: Cysteine peptidases
EDTA: Ethylenediaminetetraacetic acid
FBS: Fetal bovine serum
GP63: Zinc metalloproteinase GP63
HSPs: Heat shock proteins
IDSA: Infectious Diseases Society of America
kmp11: Kinetoplastid membrane protein 11
LCL: Localized CL
LRV1: *Leishmania* RNA Virus-1
MCL: Mucocutaneous leishmaniasis
ML: Mucosal leishmaniasis
MOI: Multiplicity of infection
mpi: Mannose phosphate isomerase
NCBI: National Center for Biotechnology Information
NO: Nitric oxide
PHOL: Public Health Ontario Laboratories
PTPs: Protein tyrosine phosphatases
RPMI: Roswell Park Memorial Institute
STPs: Serine threonine phosphatases
UDG: Uracil-DNA glycosylase
VFs: Virulence factors
