# Supplementary material for: Virulence factor RNA transcript expression in the Leishmania Viannia subgenus: influence of species, isolate source, and Leishmania RNA virus-1
Source: Trop Med Health. 2019 Apr 11;47:25. doi: 10.1186/s41182-019-0153-x (PMC6458769; doi:10.1186/s41182-019-0153-x)
Supplement: Supplementary file 1 — Supplementary Methods and Results. (DOCX 24 kb) [file 41182_2019_153_MOESM1_ESM.docx]

**Supplementary Methods**

**Macrophage Differentiation*.*** ATCC^®^ U937 CRL-1593.2^TM^ suspension cells stored in liquid nitrogen were thawed and cultured in RPMI 1640 media (Thermo Fisher Scientific, Carlsbad, CA) supplemented with 10% (v/v) heat-inactivated FBS (Thermo Fisher Scientific, Carlsbad, CA) and 1x penicillin/streptomycin (Thermo Fisher Scientific, Carlsbad, CA) at 37°C and 5% CO_2_. Cells were maintained at a concentration between 1x10^5^ and 2x10^6^ cells/mL and assessed using the trypan blue exclusion test. U937 monocytes were differentiated into macrophages by resuspending 5x10^5^ cells/ml of monocytes in RPMI-1640 supplemented with 50 ng/mL phorbol myristate acetate (PMA). One mL of monocytes were plated on removable glass cover slips onto 24-well plates and allowed to differentiate for 72 hours. Differentiated cells were identified by the presence of pseudopodia and adherence to the plate surface, while non-adherent undifferentiated monocytes were washed away with RPMI 1640 media [1-4]. Differentiated cells were released from the cover slip using 0.05% Trypsin-EDTA (Life Technologies, Carlsbad, CA, USA) and a cell count was performed.

***Leishmania* species molecular identification**. DNA was extracted using QIAamp DNA Mini Kit Blood (Qiagen, Germantown, MA, USA). *Leishmania* genus 18S real time PCR was performed as previously described [5]. Species identification included analysis of the internal transcribed spacer 1 (*ITS1*), *ITS2*, *cpb*, *hsp70*, and *mpi* targets by PCR, restriction fragment length polymorphism (RFLP) analysis, and Sanger sequencing [6-7]. PCR-RFLP analysis of the *ITS1* region can only differentiate *L. (V.) braziliensis* from the other species within the *Viannia* subgenus (*L. (V.) guyanensis, L. (V.) peruviana, L. (V.) panamensis, L. (V.) lainsoni*). Thus, PCR-RFLP and sequencing analysis of the *cpb*, *hsp70*, *mpi* and *ITS2* regions was required to differentiate species within the *Leishmania Viannia* sub-genus complex, and to provide a confirmation of the species identified in the initial *ITS1* assay. Purified PCR product was used for Sanger sequencing as per Big Dye protocol (Life Technnologies, Carlsbad, CA, USA). Sequence products were purified and analyzed using the Applied Biosystems 3130xl Genetic Analyzer. Data were standardized using the Sequencing Analyzer program and the Basic Local Alignment Search Tool (BLAST) search engine was used to analyze sequences.

**Detection and Quantification of VF RNA Transcript Expression by qPCR.** Primers were validated against Taqman^TM^ Control Genomic DNA (male) (Thermofisher Scieintific, Carlsbad, CA, USA) to ensure no cross-reactivity. In addition, primers were validated against the following ATCC® strains to ensure detection: *L. (V.) braziliensis* ATCC® 50135™ (MHOM/BR/75/M2903), *L. (V.) guyanensis* ATCC^®^50126™ (MHOM/BR/75/M4147), *L. (V.) panamensis* ATCC^®^50158™ (MHOM/PA/71/LS94), *L. amazonensis* ATCC^®^50159™ (IFLA/BR/67/PH8)*, L. chagasi* Cunha and Chagas ATCC^®^50133™ (MHOM/BR/74/PP75)*, L. donovani* (Laveran and Mesnil) Ross ATCC^®^50212™ (MHOM/IN/80/DD8)*, L. infantum* Nicolle ATCC^®^50134™ (MHOM/TN/80/IPT-1)*, L. major* ATCC^®^50122™ (MHOM/IL/67/JERICHO II)*, L. mexicana* (Biagi) Garnham ATCC^®^50157™ (MHOM/BZ/82/BEL21) and *L. tropica* (Wright) Luhe ATCC^®^50129™ (MHOM/SU/74/K27)*.*

**Supplementary Results**

**VF RNA Transcript Expression by LRV1 Status**

At 24- hours of macrophage infectivity, VF transcript expression of LRV1 positive and LRV1 negative isolates were not significantly different for *cpb* (p=0.33), *mpi* (p>0.99) and *hsp70* (p=0.67) [Figure S2]. The following targets could not be evaluated at 24-hours due to transcript levels below detection: *gp63*, *hsp23* and *hsp100*. Similarly, no significant differences were observed at 48- hours post-macrophage infection for pooled VF transcript analysis (p=0.63) , *cpb* (p=0.49) and *mpi* (p>0.99) [Figure S2]. The following targets could not be evaluated at 48- hours due to transcript levels below detection: *gp63*, *hsp23*, *hsp70* and *hsp100*.

**VF RNA Transcript Expression by Species**

Apparent increased transcript expression of *hsp100* in isolates of *L. (V.) panamensis* was noted, however this was not statistically significant (p=0.14) [Figure S3]. Pooled VF and *cpb* transcript expression was analyzed at 24- and 48- hours after macrophage infection for *L. (V.) panamensis* isolates verses other (*L. (V.) guyanensis* and *L. (V.) braziliensis*) [Figure S2]. Pooled VF transcript expression of *L. (V.) panamensis* isolates at 24- hours was significantly lower (1.31x10^6^ fold) in comparison to the other isolates (p=0.04) [Figure S4]. No difference in *cpb* transcript expression at 24- hours post-macrophage infection was observed (p=0.33). Similarly, there was no difference in pooled VF and *cpb* transcript expression at 48- hours post-macrophage infection (p>0.99) [Figure S4].

**VF RNA Transcript Expression by Source of Cultured Isolate**

Pooled VF transcript expression of ATCC and clinical strains could only be evaluated at 24- hours where a trend towards lower levels of overall VF transcript expression in clinical isolates (1.15-fold change) was observed (p=0.07) [Figure S6]. At 48- hours post-macrophage infection, VF transcript expression was not significantly different between ATCC and clinical isolates for the following: pooled VF transcript (p=0.57), *cpb* (p=0.25), and *mpi* (p=0.67) [Figure S6].

**List of Abbreviations**

phorbol myristate acetate (PMA)

internal transcribed spacer 1 (ITS1)

restriction fragment length polymorphism (RFLP)

Basic Local Alignment Search Tool (BLAST)

**References**

1. Sintiprungrat K, Singhto N, Sinchaikul S, Chen ST, Thongboonkerd (V.) Alterations in cellular proteome and secretome upon differentiation from monocyte to macrophage by treatment with phorbol myristate acetate: Insights into biological processes. J Proteomics 2010;73(3):602-18.
2. Hsiao CHC, Ueno N, Shao JQ, Schroeder KR, Moore KC, Donelson JE, Wilson ME. The effects of macrophage source on the mechanism of phagocytosis and intracellular survival of *Leishmania*. Microbes Infect 2011; 13 (12-13): 1033-44.
3. Verhoeckx KC, Bijlsma S, de Groene EM, Witkamp RF, van der Greef J, Rodenburg RJ. A combination of proteomimcs, principal component analysis and transcriptomics is a powerful tool for the identification of biomarkers for macrophage maturation in the U937 cell line. Proteomics 2004; 4(4): 1014-28.
4. Kariyawasam R, Grewal J, Lau R, Purssell A, Valencia BM, Llanos-Cuentas A, Boggild AK. Influence of Leishmania RNA Virus 1 on Proinflammatory Biomarker Expression in a Human Macrophage Model of American Tegumentary Leishmaniasis. J Infect Dis 2017; 216 (7): 877-866.
5. Wortmann G, Sweeney C, Houng HS, Aronson N, Stiteler J, Jackson J, Ockenhouse C. Rapid diagnosis of leishmaniasis by fluorogenic polymerase chain reaction. Am J Trop Med Hyg 2001;65(5):583–7.
6. Schonian G, Nasereddin A, Dinse N, Schweynoch C, Schallig HD, Presber W, Jaffe CL. PCR diagnosis and characterization of *Leishmania* in local and imported clinical samples. Diagn Microbiol Infect Dis 2003; 47: 349-58.
7. de Almeida ME, [Steurer FJ](http://www.ncbi.nlm.nih.gov/pubmed?term=Steurer%20FJ%5BAuthor%5D&cauthor=true&cauthor_uid=21752983), [Koru O](http://www.ncbi.nlm.nih.gov/pubmed?term=Koru%20O%5BAuthor%5D&cauthor=true&cauthor_uid=21752983), [Herwaldt BL](http://www.ncbi.nlm.nih.gov/pubmed?term=Herwaldt%20BL%5BAuthor%5D&cauthor=true&cauthor_uid=21752983), [Pieniazek NJ](http://www.ncbi.nlm.nih.gov/pubmed?term=Pieniazek%20NJ%5BAuthor%5D&cauthor=true&cauthor_uid=21752983), [da Silva AJ](http://www.ncbi.nlm.nih.gov/pubmed?term=da%20Silva%20AJ%5BAuthor%5D&cauthor=true&cauthor_uid=21752983). Identification of *Leishmania* spp. by molecular amplification and DNA sequencing analysis of a fragment of rRNA internal transcribed spacer 2. J Clin Microbiol 2011; 49(9): 3143-9.
