# Supplementary material for: Virulence factor RNA transcript expression in the Leishmania Viannia subgenus: influence of species, isolate source, and Leishmania RNA virus-1
Source: Trop Med Health. 2019 Apr 11;47:25. doi: 10.1186/s41182-019-0153-x (PMC6458769; doi:10.1186/s41182-019-0153-x)
Supplement: Supplementary file 2 — Figure S1. Log transformed virulence factor RNA transcript expression in baseline cultures analyzed by grouping strains according to LRV-1 status compared by Mann-Whitney for the following targets: pooled expression (A), cpb (B), gp63 (C), mpi (D), hsp23 (E), hsp70 (F), hsp90 (G) and hsp100 (H). Figure S2. Log transformed virulence factor RNA transcript expression in supernatants post-macrophage infectivity at 24 and 48 h compared by LRV-1 status using t-test for the following targets: cpb-24 h (A), mpi-24 h (B), hsp70–24 h (C), pooled-48 h (D), cpb-48 h (E), and mpi-48 h (F). Figure S3. Log transformed virulence factor RNA transcript expression in baseline cultures analyzed by grouping strains according to species (L. V. panamensis versus other) compared by Mann-Whitney for the following targets: pooled expression (A), cpb (B), gp63 (C), mpi (D), hsp23 (E), hsp70 (F), hsp90 (G) and hsp100 (H). Figure S4. Log transformed virulence factor RNA transcript expression in supernatants post-macrophage infectivity at 24 and 48 h compared by species using t-test for the following targets: pooled VF-24 h (A), cpb-24 h (B), pooled VF-48 h (C), and cpb-48 h (D). Figure S5. Log transformed virulence factor RNA transcript expression in baseline cultures analyzed by grouping strains according to source of cultured isolate (ATCC® versus clinical) compared by Mann-Whitney for the following targets: pooled expression (A), cpb (B), gp63 (C), mpi (D), hsp23 (E), hsp70 (F), hsp90 (G) and hsp100 (H). Figure S6. Log transformed virulence factor RNA transcript expression in supernatants post-macrophage infectivity at 24 and 48 h compared by source of cultured isolate using t-test for the following targets: pooled VF-24 h (A), pooled VF-48 h (B), cpb-48 h (C), and mpi-48 h (D). (DOCX 1229 kb) [file 41182_2019_153_MOESM2_ESM.docx]

**A B C D**

**E F G H**

Additional file 2

**Figure S1:** Log transformed virulence factor RNA transcript expression in baseline cultures analyzed by grouping strains according to LRV-1 status compared by Mann-Whitney for the following targets: pooled expression (A), *cpb* (B), *gp63* (C), *mpi* (D), *hsp23* (E), *hsp70* (F), *hsp90* (G) and *hsp100* (H).

**A B C**

**D E F**

**Figure S2:** Log transformed virulence factor RNA transcript expression in supernatants post-macrophage infectivity at 24- and 48- hours compared by LRV-1 status using t-test for the following targets: *cpb*-24 hrs (A), *mpi*-24 hrs (B*), hsp70*-24 hrs (C), pooled-48 hrs (D), *cpb*-48 hrs (E) and *mpi*-48 hrs (F).

**A B C D**

**E F G H**

**Figure S3:** Log transformed virulence factor RNA transcript expression in baseline cultures analyzed by grouping strains according to species (*L. V. panamensis* versus other) compared by Mann-Whitney for the following targets: pooled expression (A), *cpb* (B), *gp63* (C), *mpi* (D), *hsp23* (E), *hsp70* (F), *hsp90* (G) and *hsp100* (H).

**A B**

**C D**

**Figure S4:** Log transformed virulence factor RNA transcript expression in supernatants post-macrophage infectivity at 24- and 48- hours compared by species using t-test for the following targets: pooled VF-24 hrs (A), *cpb*-24 hrs (B), pooled VF-48 hrs (C) and *cpb*-48 hrs (D).

**A B C D**

**E F G H**

**Figure S5:** Log transformed virulence factor RNA transcript expression in baseline cultures analyzed by grouping strains according to source of cultured isolate (ATCC® versus clinical) compared by Mann-Whitney for the following targets: pooled expression (A), *cpb* (B), *gp63* (C), *mpi* (D), *hsp23* (E), *hsp70* (F), *hsp90* (G) and *hsp100* (H).

**A B**

**C D**

**Figure S6:** Log transformed virulence factor RNA transcript expression in supernatants post-macrophage infectivity at 24- and 48- hours compared by source of cultured isolate using t-test for the following targets: pooled VF-24 hrs (A), pooled VF-48 hrs (B), *cpb*-48 hrs (C) and *mpi*-48 hrs (D).
